# The Human Brain; On Disorders of the Cerebral Circulation; The Cyclopædia of Anatomy and Physiology

**Published:** 1848-04

**Authors:** 


*The Human Brain; its structure, physiology and diseases, with a description of the typical forms of Brain in the Animal Kingdom.* By SAMUEL SOLLY, F. R. S., Senior Assistant Surgeon to St. Thomas's Hospital, and Lecturer on Clinical Surgery, &c., &c. *From the Second London Edition—with one hundred and eighteen wood engravings.* 8vo. pp. 496. Philadelphia: 1848.

*On Disorders of the Cerebral Circulation; and on the connection between affections of the Brain and Diseases of the Heart.* By GEORGE BURROWS, M. D., Late Fellow of Caius College, Cambridge; Fellow of the Royal College of Physicians, London; Physician and Lecturer on the Principles and Practice of Medicine at St. Bartholomews Hospital. *With coloured plates.* 8vo. pp. 216. Philadelphia: 1848.

*The Cyclopædia of Anatomy and Physiology.* Edited by ROBERT B. TODD, M. D., F. R. S., &c., &c. *Part XXIX; containing Nervous system, physiology of the (concluded;) Pleura, and Polygastria.* 8vo. London. November, 1847.

The anatomy of the nervous system, and, still more, its physiology, have received marked attention within the last few years; and as respects the former, no one has added more to our exact information than Mr. Solly. We cannot say so much of his contributions to the physiology and pathology of the encephalon.

Twelve years ago, he published the first edition of his "*Human Brain,*" which, at that time, received merited commendations from the periodical press: now, he issues a second edition, more largely illustrated, and the American publishers have done wisely—we are sure in a scientific—and, we doubt not, in a commercial—point of view, in reprinting it, with all its riches, as it appeared on the the other side of the Atlantic.

No part of the human organism has been a subject of greater interest to the mere descriptive anatomist than the encephalon. As the organ of the wonderful functions of sensation, volition, and mental and moral manifestations, it could not fail to attract absorbing attention, and hence its various heterogeneous parts have been minutely described. Most unmeaning and absurd names were given to parts in the infancy, and even in the more advanced stages, of anatomical investigation, which have been worse than useless jargon; and have fettered the progress of the young enquirer, by loading his memory with a ridiculous nomenclature, from which every effort is now being made to free the science, and by none more than by Mr. Solly himself.

Every anatomist must have been impressed with the absurdity of calling the two forms of neurine—the *gray* or *vesicular*, and the *white* or *tubular*—the "cortical" and "medullary;" and yet these were the only names employed when *we* were taught, if we except that the gray was, at times, called "cineritious." "Cortical" was derived from *cortex* "bark," because the gray matter was seated "cortically" in the hemispheres of the brain and cerebellum; whilst the "medullary" received its appellation from being assimilated to marrow. Yet at that time it was well known that all gray matter is not placed externally; that there are masses of it seated within the hemispheres—as in the corpora striata and thalami nervorum opticorum—to use the older names—the "*anterior cerebral ganglion*" and the "*posterior cerebral ganglion*" of Mr. Solly; and that a series of ganglia extends through the very middle instead of the periphery of the medulla oblongata and medulla spinalis, the anatomical centre of the "reflex" or "excito-motory system" of Dr. Marshall Hall.

Again, the medullary portion, although assimilated by name to marrow, resembles it in no respect histologically, whilst its chemical resemblance consists in its being a phosphuretted fat, united, however, with albumen; and hence—for every reason—the vast improvement in designating the *cortical* neurine as "*vesicular*," and the *medullary* as "*tubular*;"—the very difference in structure indicating the possession of as different functional endowments;—the former destined—as we have every reason to believe—to *produce*, and the latter by its tubes to *conduct* nervous influence from one part of the economy to another; so that, by sufficient examination by the naked, and, *à fortiori*, by the assisted eye, there can be no difficulty in deciding as to the portions of the nervous system that possess the one or the other of these properties or endowments. The centre of the spinal marrow, we have seen, consists of vesicular neurine; its circumference of a sheath of tubular neurine, which is known to be continuous with the tubular matter of the brain, and in this manner prolongations of the tubular matter of the spinal marrow pass through the vesicular ganglia constituting the corpora striata and the thalami nervorum opticorum, and ultimately terminate in the vesicular neurine forming the periphery of the brain. Anatomy has shown, that the tubular neurine of the anterior portion or pyramids of the medulla spinalis, ascends through the crura cerebri and the corpora striata, whilst the posterior tubules ascend through the corpora olivaria and thalami nervorum opticorum; and as the roots of the spinal nerves, which originate from the anterior part of the spinal marrow, are known to be *motory*, and those from the posterior part *sensory*, we may thus infer, that the "anterior cerebral ganglion" (*corpus striatum*) may be connected with motion; and the "posterior" (*thalamus nervi optici*) with sensation—and such is probably the case: hence, in paralysis, the seat of the effusion of the blood—of the cerebral hemorrhage—is more frequently in the corpus striatum and its vicinity than in any other part of the brain;—the side opposite the lesion losing its motive power—as is well known—owing to the decussation of the spinal tubules at the anterior part of the medulla oblongata, and probably elsewhere, for there are many commissural arrangements.

We have alluded to ridiculous and unmeaning nomenclature; and there is not a more striking example than in the so-called "*corpora* or *tubercula quadrigemina*;" the two upper bodies of which, from their fancied resemblance to the breech, have been called *glutia* and *nates*, whilst the two lower were termed *testes*. *Now*, when we know that they are the vesicular origin of the optic nerves, what an improvement in terming them the "optic lobes or tubercles," as we term the olfactory bulbs—the olfactory lobes;—the very name conveying to the mind of the student a knowledge of their uses, whilst the ancient appellation could suggest little more than indecent associations!

As connected with this subject, we may quote with advantage the following judicious observations by Mr. Solly in his preface:—

"According to the plan generally pursued in describing the brain, in systematic works of anatomy, the information conveyed amounts to little more than a vain catalogue of names applied to parts, without reference to their structure, their functions, or even their analogies in the nervous system of the lower orders of animals. Such a barren prospect as a list of names holds out but little to attract the most zealous among students, while the dryness of unconnected detail, and the obstacles to clear conceptions engendered by the absence of everything like arrangement, almost certainly deter him from attempting to learn more than is required to prepare him for examination for the diploma. It is unfortunate, indeed, that candidates for this honourable certificate are still very generally required to describe the appearances presented by the brain dissected, or rather destroyed, by the old method of slicing; a method most unphilosophical in its conception, and totally inadequate to impart any real information in regard to the structure of the organ. And I do not hesitate to affirm that this mode of examination has contributed essentially to retard the diffusion of sound knowledge in regard to the anatomy and physiology of the most important system in the body.

"It is sad to reflect that medical students, on whom the duty devolves of tracing the relations which exist between the structure of organs and their functional manifestations, with a view to the successful treatment of disease, should thus neglect the most important part of the whole organism. No labour should be thought too great which can assist us in understanding the nature of that instrument which the mind employs in its communications with this world. Every day shows us that consciousness and volition may be disturbed by the slightest accident to the head, and that disease seldom invades the brain without dethroning the mental powers.

"When I published the first edition of this work, I was not aware that so high an authority as Dr. Craigie had exposed, in the following forcible language, the evils I then, and have since, deplored. I gladly avail myself of his authority to assist in subduing this evil:—

"'To the mind, however, which is unfettered by prejudices in favour of ancient opinions, it appears singular that the enlightened physiologists of the eighteenth century should talk of the medullary and cortical matter of an organ in which nothing like marrow or bark can be seen; and it is more extraordinary still, that the accurate distinctions which anatomy has introduced since the commencement of the nineteenth century, have not demonstrated the evil of retaining terms which are improper, as mere nominal distinctions; but which are doubly erroneous as the relics of an unfounded and exploded theory. Is the error of likening the brain to marrow, obviated by shrouding it under the learned denomination of medulla and medullary? Or is the absurdity of supposing the gray matter of the convoluted surface, a bark, or envelope to the white pith, diminished in the slightest degree by calling that gray matter cortical? The common sense of the present day will not hesitate to answer these questions in the negative.

"'Should it be said by the ambiguists, that now, when the absurdity of these names is known, it can do no harm to retain them as mere names, we answer, it may do harm; but as it can communicate no information and explain no difficulties, it is, at least, a superfluous labour to augment the confusion of a department of anatomy not very easy, by useless and antiquated names, which live only to proclaim their absurdity, and the impropriety of finding them there. Knowledge, in the present day, to be worth the labour of acquisition, ought to be accurate; the books which are to be the means of conveying this knowledge ought to contain no superfluous or erroneous information.'

"Foville, who has devoted so much attention to the structure of the brain, though he has paid too little attention to the labour of others, thus expresses himself in regard to the difficulties which attend its study;—'If we confine ourselves to the examination of its exterior, we know no more of its organization than we could know of the human body if we merely looked at the surface of the carcass: even sections of the brain teach little more than sections of the body would.'

"Foville gives to Gall the credit of teaching us how to separate the fibres of the brain without *cutting* them." p. vii.

Mr. Solly's work commences with the structural anatomy of the brain;—and this is the best portion of it. He gives its chemical constitution, the varieties of form presented by the neurine—vesicular and tubular—of animal and organic life; enters fully and clearly into the comparative anatomy, which is well elucidated by appropriate wood-cuts; ascends through the various steps of the animated ladder up to man; depicts well the protective apparatus of the human brain—*tutamina cerebri*, as they have usually been designated; gives the various weights of the brain as recorded by different observers; describes the "configuration of the encephalon;" and devotes a special "part" to the "dissection of the human brain and spinal cord"—an excellent description:—from this we extract the "Recapitulation," in which the author has indulged in further and appropriate comments on the nomenclature generally adopted in works of anatomy.

"*Recapitulation.*—The description of the course and termination of the various tracts of medullary neurine which, with their ganglia, constitute the brain or encephalon, being now concluded, it will, I think, be useful to take a general review of the subject, by a recapitulation of what has been stated in detail separately; and we will reverse the order of our observations, proceeding from above downwards, instead of from below upwards.

"In the first place, we have an extensive surface of cineritious neurine, the *hemispherical ganglion*, (speaking merely of one side of the brain,) which, in the higher orders of animals, is convoluted or folded in a peculiar manner.

"In apposition to the whole of the vesicular neurine of this ganglion, there are tubular fibres which radiate through it, and are encrusted by its nucleated cells.

"These fibres are disposed of in four different ways; 1st, some of them, commencing from the convolutions of the anterior, middle, and posterior lobes, pass through the corpora striata, and, forming the inferior layer of the crus cerebri, pass through the pons Varolii, so as to form the anterior columns of the cord, as previously described—the *motor tract:* 2d, others commencing in the nerves of sensation, and after passing through the pons Varolii, and emerging from the substance of the thalamus, terminate in the same neurine that gave origin to the last, this is the *sensory tract:* 3d, others, passing from one side of the brain to the other, and in apposition to the internal surface of all the convolutions, are those fibres which, collected into a mass, form between the hemispheres that wide bridge, if I may so call it, the *great transverse commissure*, or *corpus callosum:* 4thly and lastly, in contact with all the convolutions are the fibres of the superior and inferior longitudinal commissures, which, connecting together those convolutions which are situated on the same side of the mesial line, or different portions of the same hemispherical ganglion, so far differ from the transverse commissure, which connects those situated on opposite sides, or the two distinct but corresponding ganglia.

"The first and second set of fibres, which radiate from the external surface of the two large ganglia of the anterior and posterior columns, as from a common centre, forming, however, in their radiation, only half a circle, were designated by Gall and Spurzheim the *diverging fibres*. The third set of fibres, which converge towards the centre of the brain, the transverse commissural, were distinguished as the *converging fibres* by the same authors.

"The above descriptions demonstrate that the encephalon or brain in the human subject is not a large solid mass of matter, in the interior of which are cavities, scooped, as it were, out of its substance, to be appropriately denominated ventricles, but that it really consists of ganglia or collections of cineritious neurine, placed on each side of the mesial line. Some of them being the appropriate ganglia of the nerves of sensation; as, for instance, the olfactory ganglia, the optic ganglia or tubercula quadrigemina, the auditory ganglia or posterior pyramidal bodies, the pneumogastric ganglia or restiform ganglia, the olivary bodies or lingual ganglia; the others being the motory and sensory ganglia, as the corpora striata and thalami nervorum opticorum. The hemispherical ganglia, again, that they might present the greatest possible extent of surface, are folded up into innumerable plaits, and thus cover or surround every other ganglion within the cranium, so that on first removing the skull-cap, nothing can be seen but the convoluted surface of these extensive ganglia.

"And here let me insist upon this important principle in the study of the brain, which is also one of the first ideas that the student should acquire regarding its composition, namely, that it consists of corresponding or symmetrical parts on each side of the mesial plane, and that instead of regarding the fissures of separation between its different portions as forming ventricles or cavities, he must direct his attention to the ganglia which bound the fissure, and the structures called commissures, which, connecting them together, cross the fissure, and necessarily alter its character in different points, masking it, it is true, but not at any place changing the fissure into a true bag or circumscribed cavity. The third, the iter a tertio ad quartum ventriculum, the fourth and fifth ventricles, we have already seen, are in truth no more than the successive dilatations from below upwards of the posterior fissure of the cord; difficult enough to be understood when these are viewed in different situations and unconnected one with the other, as in the ordinary mode of dissecting the brain, but which seem necessary and obvious where its parts are traced in connection with one another.

"In conclusion, let me express the hope that these views or analyses, if I may be allowed so to call them, of the component parts of the encephalon, will really simplify the whole of its anatomy, and materially assist the student in acquiring a knowledge of its true character. I wish that custom did not require the student to burden his memory with fanciful and unmeaning names, and that, instead of learning a long catalogue of the contents of the lateral ventricles, as they are erroneously designated, and puzzling himself with the absurd titles of hippocampus major and minor, pes hippocampi, tænia hippocampi, cornu Ammonis, &c., he should be required simply to observe how the spinal columns appear to terminate superiorly in two large tubercles, the *corpora striata* and *thalami*, from the sides and under parts of which the hemispheres spring out, being afterwards reflected so as completely to envelop this bulbous extremity of the spinal cord. In the same way the third ventricle should be described as a fissure separating the two halves of the brain, his particular attention being directed to the commissures which pass across it to connect the different cerebral ganglia with one another. The description of the relative position of these ganglia, the commissures connecting them, and their relation to the ganglia and columns of the spinal cord, comprehend all the information which is either interesting or useful to the student." p. 228.

The seventh "part" or section is on the cerebral nerves; the eighth on the vessels employed in the cerebral circulation; the ninth on the development of the brain; and the tenth on the physiology of the cerebro-spinal axis. This part is exceedingly brief, and, therefore, by no means as interesting as it might have been made. It may be well, that we should epitomize what we regard as the probable functions of this the most elevated portion of the organism.

*First.* We have already observed that vesicular neurine is the source of nervous power.

*Secondly.* That tubular neurine is everywhere the conductor of nervous power.

*Thirdly.* Whenever nervous power has to be produced in greater quantity, the vesicular matter presents itself in masses or ganglia.

*Fourthly.* The three great divisions of the nervous system are: 1, The cerebro-spinal; 2, The true spinal; and 3, The sympathetic.

*a.* As regards the *cerebro-spinal* division, in which we include the vesicular and tubular matter of the brain and tuber annulare, the vesicular matter of the medulla oblongata and spinal marrow, and in which are the organs of sensation, volition, and mental and moral manifestation.

In this division, we meet with numerous parts. There is great reason to believe, that the *vesicular matter of the cerebrum* occupying the convolutions and anfractuosities is mainly concerned in the production of the mental and moral manifestations,—that the *corpora striata* and *thalami nervorum opticorum*—in other words, the anterior and posterior cerebral ganglia of Mr. Solly—are sensori-motor centres, as before remarked. The *locus niger*, in the crus cerebri, is considered by our author to be the "serial hemologue and analogue" of the anterior peaks of gray matter of the spinal cord, and is, he supposes, the seat of the excito-motor power of the third pair of nerves. The functions of the *optic lobes or tubercles* we have referred to already; and there is great ground for according with the facts and arguments adduced by Dr. Carpenter, that a series of *sensory ganglia* exists at the base of the brain, which may be regarded as the special encephalic organs of the senses;—and that these may be present in animals not provided with cerebral hemispheres, and whose actions are consequently entirely sensori-volitional; but whether they are the nervous seat of the emotional and of all the instinctive acts admits of great question, as we may take occasion to show hereafter, when more time and space are permitted us.

We have remarked, that the nervous seat of intellect is probably the vesicular matter of the cerebral hemispheres; yet we are not prepared to subscribe to the doctrine, that separate and distinct organs, the seat of as many special and distinct primary faculties, exist there—as maintained by the phrenologists; and we may say further, that year after year's observation satifies us more and more of the difficulties in the way of such a conclusion; and that the most cogent facts and arguments may be brought forward against the location even of the instinct of generation and of philoprogenitiveness—without the existence of which many animal species would necessarily cease—and the assigned phrenological seats of which have generally been regarded as most strongly established.

The *cerebellum* appears to be connected with the regulation and co-ordination of the movements; and, most assuredly, as we descend the scale of animals, the prominences of the skull caused by the posterior portions of the hemispheres, in which the organ of philoprogenitiveness is placed—soon disappear, and are not indicated externally in animals, which, notwithstanding, are marked for the intensity of their maternal instinct.

*b.* The true *spinal* nervous system—the second division—is seated in the vesicular matter within the medulla spinalis, which may be regarded as a series of ganglionic centres, "structurally homologous and functionally analogous to the jointed ganglionic cord of the articulata, and although we are unable to point out any corresponding lines of demarkation between them, they are as functionally distinct as the auditory, optic, and olfactory ganglion of the brain." In the vesicular ganglia in the centre of the medulla oblongata, we have the nervous centre of respiration and deglutition; and in that throughout the medulla spinalis, including the medulla oblongata, we have the nervous system of involuntary actions, with which afferent and efferent nerves communicate,—in the aggregate constituting the *reflex* or *excito-motory system* of Dr. Marshall Hall.

c. The *sympathetic*—as its name imports—appears to be the portion of the nervous system, which is the great medium of association and sympathy; and by which the functions of secretion and nutrition especially are maintained in appropriate consent.

Such is an imperfect epitome of what appear to be the functions of the various parts that collectively constitute the nervous centres.

The concluding part of Mr. Solly's work is on "*Diseases of the Brain*," which he divides into—1. Anæmic affections; 2. Hyperæmic affections; 3. Convulsive affections; and 4. Organic affections; an arrangement which, as he properly remarks, is like every other that has been adopted, of course liable to objections, as they may all run into each other, so that the lines of distinction may be lost; nevertheless, in a practical point of view, he hopes "it will on the whole, be found advantageous."

We cannot dwell upon this portion of the volume. It appears to contain not a very comprehensive account of the various cerebro-spinal affections; and nothing that strikes us as adding materially to our stock of pathological or therapeutical knowledge. Still, we can strongly recommend the whole work to the student, young and old, for rich and rare information touching a most important part of the organism in its healthy and diseased condition.

The work of Dr. Burrows takes a more limited range than that of Mr. Solly. It is restricted, as its title imports, to "Disorders of the Cerebal Circulation," &c. The views of the author, in regard to the varying quantity of blood in the cranium, have often been referred to in the Journals and in the standard treatises on the Practice of Medicine. They ought, indeed, to be familiar to every practitioner, in consequence of the erroneous physiology and pathology, tending to equally erroneous therapeutics, which have been strongly urged by Monro the second, Drs. Abercrombie, Kellie, and Clutterbuck, amongst others. To Dr. Burrows—as Mr. Solly has remarked—belongs the credit of dispelling these illusions. Resting chiefly on the recorded results of certain experiments by Dr. Kellie, of Leith, which were published in the first volume of the "Transactions of the Medico-Chirurgical Society" of Edinburgh, many modern physiologists have maintained, that the quantity of blood in the cranium never varies, and that the brain is incompressible; and under this notion, Dr. Clutterbuck affirmed "that no additional quantity of blood can be admitted into the vessels situated in the brain, the cavity of the skull being already completely filled by its contents. A plethoric state or overfulness of the cerebral vessels altogether, though often talked of, can have no real existence; nor, on the other hand, can the quantity of blood within the vessels of the brain be diminished; no abstraction of blood, therefore, whether it be from the arm, or other part of the general system, or from the jugular veins, (and still less from the temporal arteries,) can have any effect on the blood-vessels of the brain, so as to lessen the absolute quantity of blood contained in them."

It was important, then, to establish the truth or inaccuracy of these views, which Dr. Burrows has done most conclusively by experiments on animals. From the results of these experiments, he infers, that it is not a fallacy, as some suppose, that bleeding diminishes the actual quantity of blood in the cerebral vessels. By abstraction of blood we not only diminish the momentum of blood in the cerebral arteries and the quantity supplied to the brain in a given time, but we actually diminish the quantity of blood in these vessels. "Whether the vacated place is replaced by serum or resiliency of the cerebral substance under diminished pressure, is another question into which," he says, "I do not now enter."

Dr. Burrows likewise institutes facts and arguments to decide, whether position could affect the condition of the vessels within the skull; as Dr. Kellie had asserted, that the quantity of blood in the cerebral substance is not modified by posture. Two full grown rabbits were killed by hydrocyanic acid, and whilst their hearts were still pulsating, one was suspended by the ears; the other by the hind legs. They were left so suspended for twenty four-hours; and before they were taken down for examination, a tight ligature was placed round the throat of each rabbit, to prevent, as effectually as possible, any further flow of blood to or from the head, after they were removed from their respective positions. The contrast in the appearances presented by the two animals, was most striking. "In the one was to be seen a most complete state of anæmia of the internal as well as external parts of the cranium; in the other a most intense hyperæmia or congestion of the same parts; and these opposite conditions in the vascularity of the brain induced solely by posture and the gravitation of the blood." The appearances presented in the two cases are represented by lithographic illustrations in Dr. Burrows's work, which we can recommend to the careful perusal of every observer.

The same want of space which occasions us to treat so briefly of Dr. Burrows's excellent production, will enable us only to say, that in the 29th part of the "Cyclopædia of Anatomy and Physiology," which comes to us ever and anon, like 'angel's visits,' we have the termination of a good view of the physiology of the nervous system by Dr. Todd,—not orthodox, according to our views, in all respects, but still an able and excellent essay of the subject.

